# Analysis of the Metals in Soil-Water Interface in a Manganese Mine

**DOI:** 10.1155/2015/163163

**Published:** 2015-06-17

**Authors:** Bozhi Ren, Qian Wang, Yangbo Chen, Wenjie Ding, Xie Zheng

**Affiliations:** College of Civil Engineering, Hunan University of Science & Technology, Xiangtan, Hunan 411201, China

## Abstract

In order to reveal the influence of the metals of soil-water interface in a manganese mine (Xiangtan, China), on local water environment, there are six kinds of metals (Mn, Ni, Cu, Zn, Cd, and Pb) characterized by measuring their concentration, correlation, source, and special distribution using principal component analysis, single factor, and Nemero comprehensive pollution index. The results showed that the corresponding average concentration was 0.3358, 0.045, 0.0105, 0.0148, 0.0067, and 0.0389 mg/L. The logarithmic concentration of Mn, Zn, and Pb was normal distribution. The correlation coefficients (between Mn and Pb, Mn and Zn, Mn and Ni, Cu and Zn, Cu and Pb, and Zn and Cd) were found to range from 0.5 to 0.6, and those between Cu and Ni and Cu and Cd were below 0.3. It was found that Zn and Mn pollution were caused primarily by ore mining, mineral waste transportation, tailing slag, and smelting plants, while Cu and Ni mainly originate from the mining industry activities and the traffic transportation in the mining area. In addition, the Cd was considered to be produced primarily from the agricultural or anthropogenic activities. The pollution indexes indicated that metal pollution degree was different in soil-water interface streams as listed in increasing order of pollution level as Zn > Ni > Cu > Pb > Mn > Cd. For all of the pollution of the soil-water interface streams, there was moderate metal pollution but along the eastern mine area the pollution seemed to get more serious. There was only a small amount of soil-water interface streams not contaminated by the metals.

## 1. Introduction

For long-term mining and metallurgy, a large amount of manganese as well as the associated metals (Ni, Cu, Zn, Cd, Pb, etc.) enters into the mine soil through different ways. Thereafter, the rainfall runoff can make the metal stream pollution formed in soil-water interface, which is a special form of nonpoint source pollution [1–3]. If the stream pollution migrates to mining area and surrounding environment along with surface runoff, it can cause the metal pollution to local water environment. However, research on the stream source and level of the pollution is meeting a challenge because, in a manganese mine, the topographical and geological conditions are relatively complex, and the solid waste (such as ore, waste ore, tailings, and slag), transportation, and production wastewater in this area are larger and distributed widely. This increases the difficulties in monitoring, control, and management of the metal stream pollution [4–8].

The metal stream pollution in a manganese mine located in Xiangtan, China, called Hongxin manganese mine, was investigated in this study. The characteristics of the metals (Mn, Ni, Cu, Zn, Cd, and Pb) including the concentration, source apportionment, and the correlation between metal elements were examined via the geostatistical methods in combination with ArcGIS 9.3 software. In addition, the special distribution of the metal pollution was assessed via Nemero comprehensive pollution index, which provides a profoundly scientific guidance on evaluation and control, management, and restoration of the metal pollution for manganese ore and other mining areas.

## 2. Monitoring Site and Sampling

### 2.1. Monitoring Site

The investigated Hongxin mine in this study is about 12 kilometers north of Xiangtan with longitude between 111°58′ and 113°05′E and latitude between 27°21′ and 28°05′N. There is a dry summer and autumn with annual average temperature between 16.7 and 17.4°C, whereas annual rainfall is often adequate up to about 1300 mm and it varies with season. In northern, southern, and western parts there is a higher terrain but it is lower in the central and eastern parts. Overall, the terrain is relatively smooth. The mine used to be called “Manganese Capital” because there are numerous coalmine, quarry, smelter, and electrolytic plants, abandoned plants, residential areas, solid waste, and so forth. For approximately hundred years of mining, the geological environment and the ecological environment have been damaged seriously so that the mining ground collapses, and the solid wastes (waste rock, coal gangue, and tailings) lumbers and vegetation have also been damaged; the soil has been eroded; water environment has been contaminated by the metals, and so forth, which have seriously hindered sustainable development of the manganese ore zone economy.

### 2.2. Sampling

**Figure 1:**
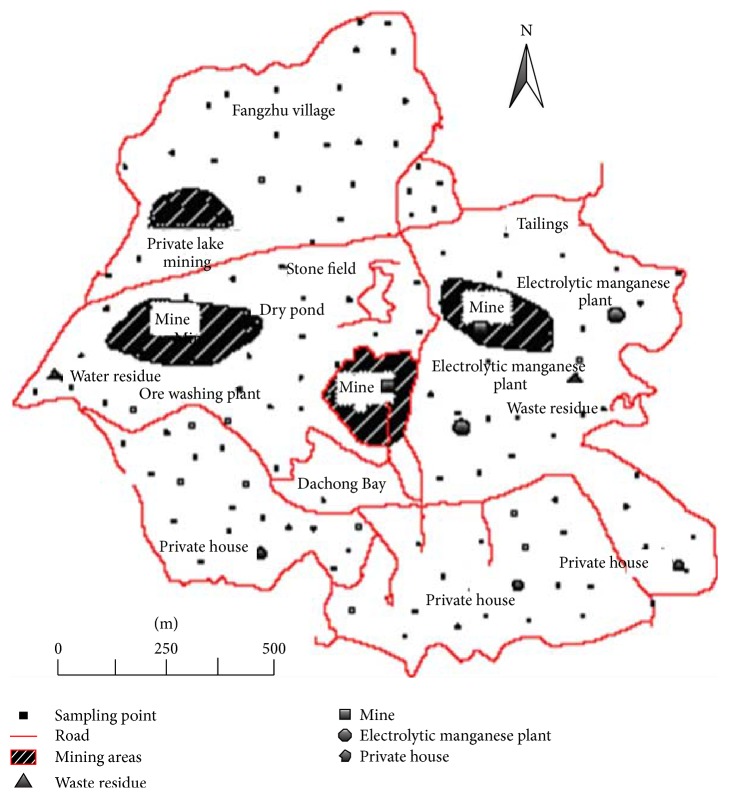
Map of monitoring sites.

In this study, there were 120 sampling points distributed evenly in the investigated area with the sample interval of 0.02 km^2^ as shown in Figure 1. It shows the map of sampling points distribution in which the road, mining areas, waste residue sites, and so forth were labeled. There were four mining areas labeled in this map. Those monitoring sites were around the mining area. Therefore, the mining activates were bound to have a significant influence in the environment of the monitoring sites. Under natural rainwater conditions, the surface runoff could be generated based on the fact that the rainwater intensity was greater than the infiltration intensity. At each sample point, 0.5–3 L of surface runoff water samples was collected and transferred to the corresponding polypropylene container. The polypropylene container was thoroughly rinsed and dried prior to use. The sampling time was recorded along with the latitude and longitude of the sampling points collected by a scientific GPS instrument. Subsequently, the container was packed in a black plastic bag with ice ensuring the freshness of the water samples and taken to laboratory for further processing within 24 hours.

There are 32 monitoring points data to be selected for data analysis in laboratory. The concentrations of the metals of the samples were measured using Chinese National Standard for drinking water testing (no. GB5750.6-2006). A brief description about this method was that the sample was firstly shaken prior to the settlement, followed by a natural settlement of 30 min. After the settlement, the supernatant was siphoned and filtered through 0.45 *µ*m membrane. Finally, the pH of the sample was lowered to < 2 by adding nitric acid (HNO_3_) to the filtered sample and the sample after acidification was stored in the dark at 4°C for later analysis. The metals (Mn, Cu, and Zn) were measured via flame atomic absorption spectrometry (AA7003A, Dongxi Research Institute of Electronics Technology, China). Others (Ni, Pb, and Cd) were measured using the graphite furnace atomic absorption spectrometry. The data analysis was carried out via a scientific software (SPSS19. 0) [9] in order to obtain the characteristics of the metals pollution.

### 2.3. Source Analysis and Pollution Evaluation

There were six kinds of metals (Mn, Ni, Cu, Zn, Cd, and Pb) to be analyzed in this study where the correlation between the metals and the principal component analysis methods were adopted. The correlation degree of the metals was expressed with the correlation coefficients calculated by the linear fitness. The correlation coefficient value ranged between −1 and 1 [10, 11]. When the correlation coefficient is closer to 1, it showed a strong correlation between the metals. Therefore, it can indicate the difference in correlation between metals concentration. The principal component analysis (PCA) used in this study was to analyze which metals were the main pollution source. It is such a statistical method that uses an orthogonal transformation to convert a set of observations of the potentially correlated variables into a set of values of linearly uncorrelated variables [12]. This method is able to show contribution rate, accumulative contribution rate, screen plot, loading matrix, and the three-dimensional factor loading graph [13–16]. In addition, the widely used single factor and Nemero comprehensive index in combination with ArcGIS software were used to calculate the relevant parameters and complete the interpolation analysis by Craig function [17] and subsequently to evaluate the metals pollution thus presenting special distribution of the pollution index while reflecting pollution level of rainwater runoff [18–25]. There are two typical indexes having been widely used, including single factor index and the comprehensive pollution index [19, 26–28]. They can be calculated through different mathematical models to determine the pollution degrees. In this study, the single factor (see (1)) and Nemero comprehensive index (see (2)) were expressed by the following forms:

(1) Pi=CiSi

(2) P=1n∑i=1nP2+Pimax2,

where *C*_*i*_ is measured value for the *i*th metal, *P*_*i*_ is single factor pollution index for the *i*th metal, *S*_*i*_ is pollution assessment standard (mg/L), *P* is comprehensive pollution index, and *n* is total number of the evaluated metals.

According to Chinese water quality standards (GB/T 3838-2002) for surface water, the standard value of water quality of class I was selected as the reference index of the metal stream pollution evaluation, and the pollution level was classed into four types according to *P*_*i*_, *P* values (*P*_*i*_ ≤ 1 as no pollution, 1 < *P*_*i*_ ≤ 5 and 1 < *P* ≤ 3.6 as light pollution, 5 < *P*_*i*_ ≤ 10 and 3.6 < *P* ≤ 7.1 as moderate pollution, and *P*_*i*_ > 10 and *P* > 7.1 as severe pollution). The metals concentration in this study was selected as main pollution indicators.

## 3. Results and Discussion

### 3.1. The General Characteristics of Metal Pollution in Runoff in Manganese Ore Area

**Figure 2:**
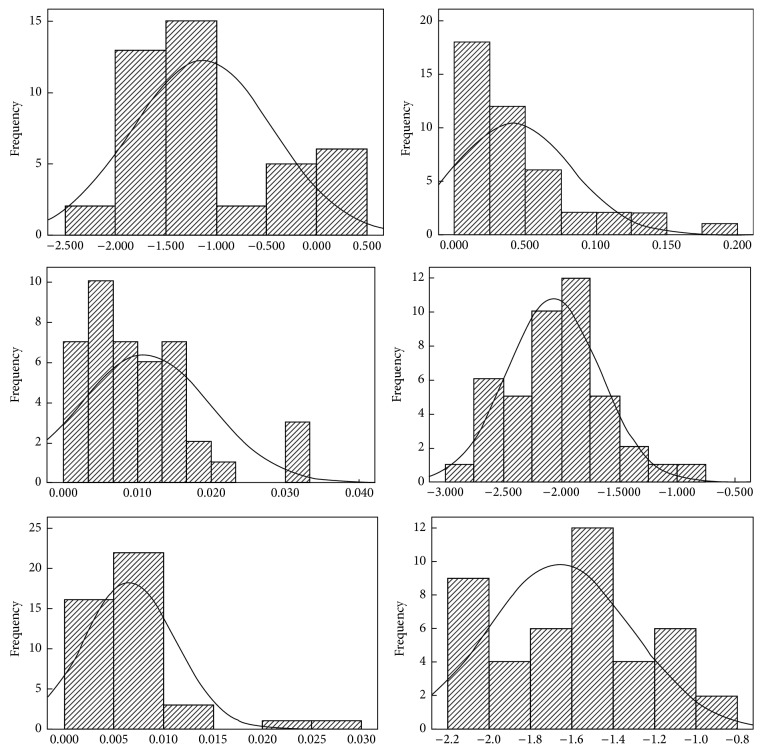
The metals frequency.

The statistical analysis of the metals concentration including Mn, Ni, Cu, Zn, Cd, and Pb is shown in Table 1 and Figure 2. The coefficient of variation of each sample could reflect the average degree of variation of the total samples. The coefficient of kurtosis reflects top sharpness of curve and flat degree. The coefficient of skewness is to reflect the symmetry of curve. As shown in Table 1 and Figure 2, the corresponding metal average concentration was 0.3358, 0.045, 0.0105, 0.0148, 0.0067, and 0.0389 mg/L. The difference between the maximum and the minimum metal concentration values was relatively higher, which had an accumulation effect of the metals. This possibly posed a threat to environment. The average variation degree was listed in decreasing order as Mn > Zn > Ni > Pb > Cd > Cu. The Mn and Zn had a stronger variation because their coefficients of variation were up to 1.88 and 1.35, respectively. For Ni, Pb, Cd, and Cu, their coefficients were up to between 0.5 and 1. The metal concentration (Mn, Zn, and Pb) was logarithmic normal distribution while those of Ni, Cu, and Cd were normal distribution. These variations were possibly attributed to the complexity of geological environment and mining behaviors, smelting and processing, solid waste (ore, waste ore, tailings sand, and slag), transportation, and other human activities [29, 30].

### 3.2. Source Analysis of the Metals

#### 3.2.1. Correlation Analysis

The analysis of correlation between the metals indicated that the source of the metals can be traced, which determine whether they come from the same source. The analytical results were shown in Table 2. A strong correlation would indicate that they would come from the same pollution source and that their formations were possibly caused by the natural or industrial production or the human activities. Otherwise, they would come from different pollution sources, and their formations were generated through other pathways. The results showed that Mn and Zn had better correlation with other metals. The correlation coefficients (between Mn and Pb, Mn and Zn, Mn and Ni, Cu and Zn, and Cu-Pb and Zn-Cd) were between 0.5 and 0.6; thus, they had higher possibilities coming from the same pollution source. On the contrary, because the correlation coefficients (between Ni and Pb, Ni and Cu, and Cu and Cd) were less than 0.3, it would indicate that they might come from different pollution sources. Ni had higher correlation with Mn but lower with the other metals, which suggested that Ni was possibly produced from other pollution sources. The significant difference in the correlation of those metals identified in this study was exactly helpful for us to analyze their pollution sources, especially in those of Mn and Zn.

#### 3.2.2. The Principal Component Analysis

**Figure 3:**
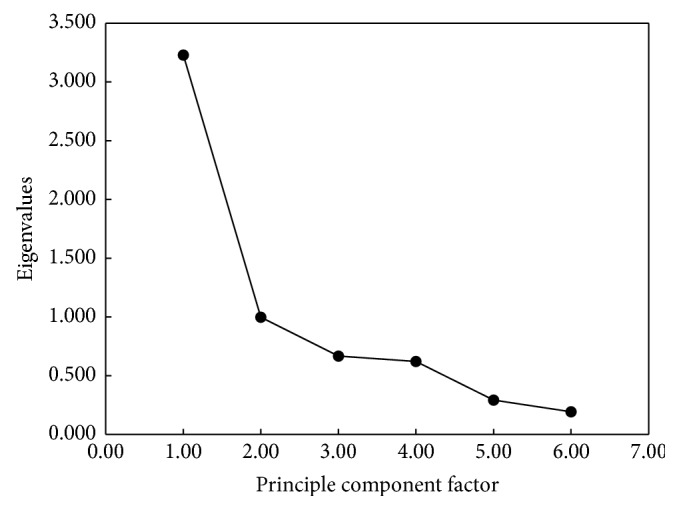
Scree plot.

**Figure 4:**
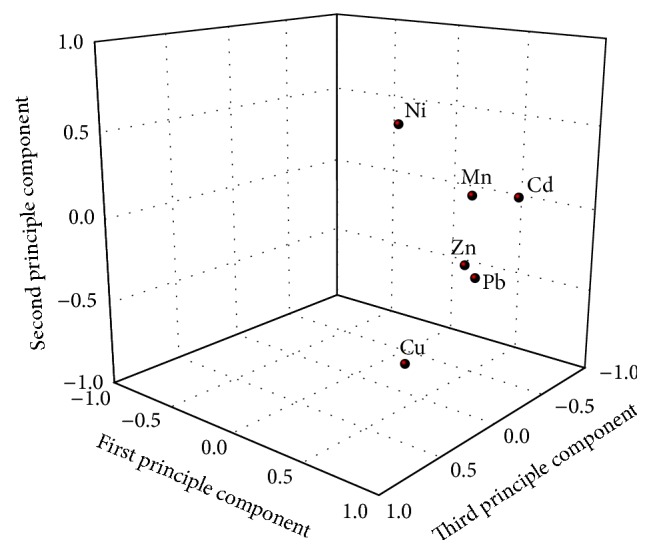
Three-dimensional loading plots of heavy metals.

The principle component analysis was carried out to obtain the major metal pollution components via SPSS 19.0 software. The analysis was assessed by measuring the contribution rate, the accumulative contribution rate, and the principal component scree plot as shown in Table 3 and Figure 3, respectively. The loading matrix of the principal components and three-dimensional factor of loading graph are shown in Table 4 and Figure 4, respectively.

As seen from Figure 3, it can be found that there was a steep scree graph, which indicated that the differences between factors were larger, and the first three principle components reflected the most of the whole samples information; thus they can be extracted and used for representing the whole information of samples sources. As shown in Figure 4 and Table 4, six principle components were obtained and the accumulative contribution rates of the first three components were up to 81.5%, which satisfied basic requirement of principal component extraction. The first principle component having the maximum contribution rate could explain 53.8% of source information of the metals, which was mainly composed of Mn and Zn and others partially coming from Pb, Cd, Cu, and Ni. The second principle component can explain 16.6% of the whole samples source information. Ni and Cu had higher loading while the loadings of the left four elements were lower, and even they almost had no relevance to Pb, Zn, and Mn. According to the correlation analysis, the loadings of Mn, Zn, and Pb were higher in the first principle component and Mn, Zn, and Pb were positively related to the other metals, which indicated that one or several of them would come from the same pollution source. According to the actual pollution distribution status of the investigated area and field survey, the metals pollution was produced mainly from the ore mining, ore material transport, waste slag of tailings, leachate, and smelting plants. In the second principle component, the loadings of Cu and Ni were relatively higher, which indicated that the actual role of the contribution rate of the second principle component was significant. Because the relative correlation between Cu and Ni was lower, their sources would be different. Among them, Cu mainly came from industrial pollution such as smelting industry and industrial waste. Because the study area is located in the rural town and transportation runs crossing the whole study area, thus the traffic would be the major source of Ni emission. Therefore, the second principle component suggested that the metal pollution to the study area be caused by the industrial activities and transportation. The third principle component had higher loadings of Cd; Zn and Mn had higher correlation with Cd but their loadings were lower, which indicated that Cd might have other sources. Cd is often produced from smelting industry, which is easily absorbed by crops, and pollutes runoff through wastewater and waste slag. In addition, Cd resulting from the vessel pipeline pollution can also pollute the drinking water. Therefore, the third principle component can indicate the pollution to environment caused by the agricultural activities and human activities such as sewage irrigation and living garbage.

### 3.3. Assessment of the Metals Pollution

**Figure 5:**
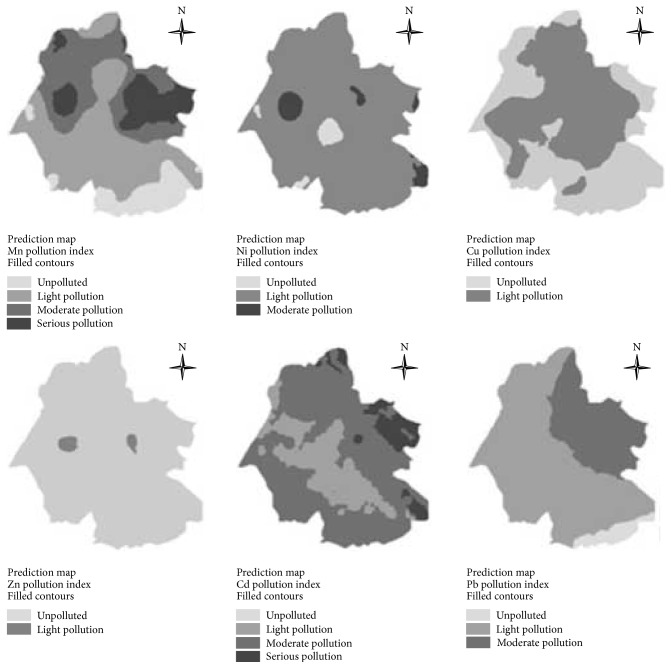
Distribution maps of six kinds of metal pollution indexes.

According to the standard method, the metals' single factor, *P*_*i*_, and the comprehensive pollution index, *P*, were analyzed. The statistical results were shown in Table 4. According to the spatial coordinates of sampling points and the statistical analysis theory, the Kriging interpolation method was used to determine the special distribution of the *P*_*i*_ and *P* values of the metals via ArcGIS. The Kriging interpolation is such a method of interpolation for which the interpolated values are predicted [31]. Their special distribution was plotted as shown in Table 5 and Figure 5.

As seen from Table 5, for the 43 water samples, there were 30 water samples unpolluted by Mn and among them 3 water samples were contaminated mildly and 4 moderately polluted and 6 seriously polluted. There are 13 water samples not subjected to Ni pollution, 26 water samples contaminated by Ni lightly, and 4 water samples moderately contaminated by Ni. 24 water samples were not subjected to copper pollution, 19 water samples were contaminated by Cu lightly, 40 water samples were not subjected to zinc pollution, and 3 water samples were contaminated by Zn slightly. There were 5 water samples not contaminated by cadmium, 11 mildly polluted, 22 moderately polluted, and 5 seriously polluted by Ca. There were 9 water samples not contaminated by Pb, 22 water samples subjected to mild Pb pollution, 10 water samples subjected to moderate Pb pollution, and 2 water samples subjected to serious Pb pollution. The comprehensive pollution indexes of 43 samples showed that there were 10 water samples polluted lightly, 23 water samples subjected to moderate pollution, and 10 water samples being seriously polluted. The results of the pollution index assessment shown in Table 5 indicated the pollution degree of the water samples by the metals according to the *P*_*i*_ and *P* values. From the analysis of the results obtained, the used method in this study was effective for evaluation of the metal pollution distribution.

**Figure 6:**
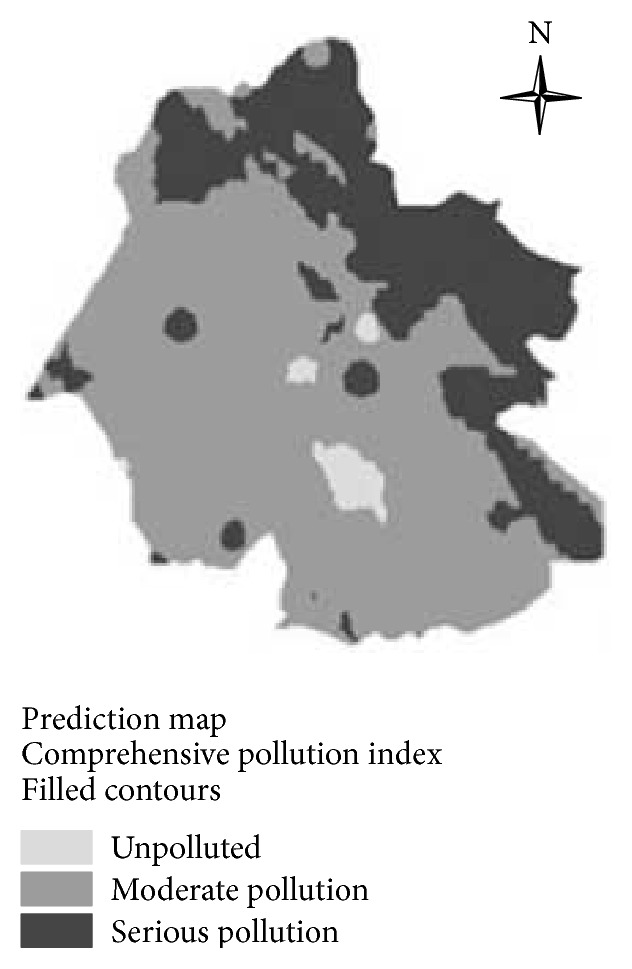
Distribution maps of comprehensive pollution indexes.

Figure 5 showed that, in northern part of the study area, the soil-water interface streams were polluted moderately or seriously in most of the regions. On the contrary, in the southern part of the study area, the soil-water interface streams were polluted by Mn slightly in most of the regions. In most of the regions, the soil-water interface streams were mildly polluted by Ni, and small amounts of the regions were influenced moderately. There were about half of the regions where the soil-water interface streams were contaminated by Cu mildly. In a small area, the soil-water interface streams were contaminated by Zn mildly or no pollution to the streams was found. In addition, there was common Cd pollution. However, in the eastern parts of the study area, there was serious Cd pollution. From the perspective of environmental risk and human health, Cd should be required prior to be governed and controlled. In the 2/3 study area, there was mild Pb pollution in the soil-water interface streams, and in the northeast there was moderate Pb pollution.  Figure 6 showed that the soil-water interface streams were contaminated by metal to varying degrees. In most of the study regions, there existed moderate metal pollution, whereas along the eastern area, the metal pollution was more serious. There were only few areas unpolluted. Therefore, the metal pollution of the coal areas to the environment needs to be urgently controlled, especially for those seriously polluted areas.

## 4. Conclusions

In this study, the metals in the soil-water interface in a manganese mine were analyzed with the statistical methods. From above discussions, we come to the following conclusions.

1. The difference in the content of the metals in soil-water interface streams in a manganese mine was very significant. They changed significantly and their pollution distributions were uneven. The coefficients of variation of Mn and Zn could reach 1.88 and 1.35. The frequency curve coefficient of kurtosis of each metal was relatively larger, while the skewness coefficient was lower. They showed a strong regional difference.
2. The correlation coefficients of the metals (between Mn and Pb, Mn and Zn, Mn and Ni, Cu and Zn, Cu and Pb, and Zn and Cd) in the soil-water interface streams pollution were between 0.5 and 0.6. This indicated that the possibilities of pollution of several metals from the same pollution sources were higher. The correlation coefficients of other metals (between Cu and Ni and Cu and Cd) were less than 0.3, which indicated that these metals come from different sources. The correlations of Ni with other metals except with Mn were lower. This implies that Ni would come from different sources compared with other four metals.
3. The first principle component of the metals stream pollution of soil-water interface in manganese mine can explain 53.68% of the components information, and six kinds of metals had higher positive loadings, which indicated that these metals came from the mining activities as a major factor of metal pollution. The ore mining, mineral aggregate transport, tailings waste, leachate, and smelter would be the main source of causing the pollution of Mn, Zn, and Pb. In the second component, Cu and Ni had higher loadings, which were the source of moderate metal pollution by the industrial activities and the traffic transportation. The third principal component was dominated by the metal, Cd, which implied the light metals pollution coming from the agricultural activities and the human activities.
4. The soil-water interface streams were seriously contaminated by Cd, followed by Mn and Pb. Some metals (Zn, Cu, and Ni) had less pollution to the interface streams. The spatial distribution of six kinds of metals pollution index and comprehensive pollution index showed that there was a moderate metal pollution to the storm water runoff in most of the manganese area; along with the eastern area, there was serious metal pollution to storm water runoff; there was less area not affected by the metal. The degree of the metal pollution to the soil-water interface streams was listed here in increasing order of effectiveness as Zn > Ni > Cu > Pb > Mn > Cd. From the environment and human health risk perspective, Cd should be given priority treatment and control.

Overall, the above results were able to show the metal pollution distribution within the investigated areas. It is significant for assessment of environmental risk of the metal pollution to the mine area, and it can give a technical support of making a decision on monitoring and controlling metal pollution for the environmental manager, government, and other related departments.

## Figures and Tables

**Table 1:** Statistical characteristics of the metals concentrations.

Metal	Mn	Ni	Cu	Zn	Cd	Pb
Minimun (mg/L)	0.0049	0.0023	0.0014	0.0014	0.0002	0.0074
Maximum (mg/L)	3.0419	0.1932	0.0313	0.1124	0.0281	0.1269
Mean (mg/L)	0.3358	0.045	0.0105	0.0148	0.0067	0.0389
Std. (mg/L)	0.634	0.041	0.0076	0.02	0.005	0.032
Coefficient of variation	1.88	0.91	0.72	1.35	0.75	0.82
Coefficient of skewness	2.716	1.725	1.365	3.459	2.094	1.275
Kurtosis	8.117	3.213	1.772	13.426	6.891	1.013
Distribution types	Logarithmic normal distribution	Normal distribution	Normal distribution	Logarithmic normal distribution	Normal distribution	Logarithmic normal distribution

**Table 2:** Correlation test of the metals.

Correlation coefficient	Mn	Ni	Cu	Zn	Cd	Pb
Mn	1					
Ni	0.576^*∗∗*^	1				
Cu	0.342^*∗*^	0.119	1			
Zn	0.578^*∗∗*^	0.436^*∗∗*^	0.609^*∗∗*^	1		
Cd	0.499	0.307^*∗*^	0.250	0.578^*∗∗*^	1	
Pb	0.605^*∗∗*^	0.251	0.513^*∗∗*^	0.434^*∗∗*^	0.456^*∗∗*^	1

*Note*. *∗∗* stands for significance (*P* < 0.05); *∗* stands for significance (*P* < 0.01).

**Table 3:** Principal component analysis of the metals concentrations.

Principle component	1	2	3	4	5	6
Eigenvalues	3.228	0.998	0.667	0.622	0.294	0.192
Contribution rate (%)	53.800	16.629	11.115	10.362	4.892	3.203
Cumulative contribution rate (%)	53.800	70.428	81.543	91.905	96.797	100.000

**Table 4:** Content loading matrix of the metals.

Metal	Mn	Ni	Cu	Zn	Cd	Pb
Component						
1	0.832	0.595	0.641	0.838	0.711	0.750
2	0.264	0.661	−0.636	−0.102	0.101	−0.255
3	0.037	0.367	0.340	0.095	−0.622	−0.141

**Table 5:** Evaluation of the metal pollution indexes.

Metal	Pollution index							
	*P*_*i*_ ≤ 1	*P* ≤ 1	1 < *P*_*i*_ ≤ 5	1 < *P* ≤ 3.6	5 < *P*_*i*_ ≤ 10	3.6 < *P* ≤ 7.1	*P*_*i*_ > 10	*P* > 7.1
Mn	30	0	3	10	4	23	6	10
Ni	13		26		4		0	
Cu	24		19		0		0	
Zn	40		3		0		0	
Cd	5		11		22		5	
Pb	9		22		10		2	
